# The Use of Mobility Devices and Personal Assistance: A Joint Modeling Approach

**DOI:** 10.1177/2333721419885291

**Published:** 2019-10-25

**Authors:** Hongdao Meng, Lindsay J. Peterson, Lijuan Feng, Debra Dobbs, Kathryn Hyer

**Affiliations:** 1 University of South Florida, Tampa, USA; 2 Peking University, Beijing, China

**Keywords:** assistive device, mobility, personal assistance, disability, accommodation

## Abstract

**Objective:** To examine whether mobility device use substitutes for personal assistance among U.S. older adults. **Method:** Using the National Health and Aging Trends Study, we identified 3,211 community-living older adults (aged 65 and older) who reported mobility difficulties at baseline. We used recursive bivariate probit models to simultaneously estimate the effect of covariates on the likelihood of using (a) mobility devices and (b) personal assistance to accommodate mobility difficulty. Independent variables included age, gender, race, physical/mental health status, cognition, and comorbidities. **Results:** Predictors of the use of personal assistance and mobility devices exhibit important similarities and differences. Device use reduced the odds of receiving personal assistance by 50% (odds ratio [OR] = 0.50, 95% confidence interval [CI] = [0.29, 0.86]). **Discussion:** Findings suggest device use substitutes for personal assistance. Practitioners and policymakers should promote the appropriate use of mobility devices while recognizing the importance of assistance with some groups and the potential of increasing mobility device use.

## Introduction

Mobility, the ability to move from place to place for the completion of daily tasks, is an essential component of quality of life among older adults. Mobility limitation is often one of the early signs of functional decline and an important component of frailty (Fried, Young, Rubin, Bandeen-Roche, 2001). Individuals with mobility limitations are more likely to be sedentary, to limit social contact, and to experience chronic conditions such as obesity, cardiovascular disease, diabetes, poor cognitive function, and depression (Bohannon, 2011; Rosso, Taylor, Tabb, & Michael, 2013; Saajanaho et al., 2016). For these individuals, compensation for the mobility limitations by using devices (cane, walker, wheelchair, and scooters) and/or personal assistance is important in maintaining quality of life and social engagement (Freedman, Kasper, & Spillman, 2016; Giesbrecht, Smith, Mortenson, & Miller, 2017).

Studies have shown that the use of mobility devices can increase physical stability, confidence, and independence (Brown & Flood, 2013; Resnik, Allen, Isenstadt, Wasserman, & Iezzoni, 2009); therefore, the use of assistive devices of all types has been rising for more than a decade (Spillman, 2005; Spillman, 2014). However, a recent national survey found that 29% of U.S. adults 65 and older, or 11 million people, received personal assistance because of an activity limitation (Freedman & Spillman, 2014).

Although both devices and personal assistance enable individuals to accommodate their activity limitations, researchers have focused on the greater potential of device use to enable older adults to age in place with independence and to ease pressure on family caregivers and a strained long-term care workforce. However, to date, evidence is mixed about the extent to which assistive devices are being used as substitutes for personal help. Studies have shown that basic mobility devices, such as canes, can reduce the need for personal help (Agree & Freedman, 2000; Allen, Foster, & Berg, 2001). Others found that device use was associated with fewer hours of help for functional limitations (Hoenig, Taylor, & Sloan, 2003). However, Agree, Freedman, Cornman, Wolf, and Marcotte (2005) found that devices did not substitute for personal care for most older adults living in the community. Rather they were associated with a higher probability of receiving personal care and using more caregivers and more care hours. Anderson and Wiener (2015) found evidence that mobility devices partially substituted for personal assistance. Therefore, the potential substitution effect of mobility device use on personal assistance remains inconclusive.

The objective of the present study was to examine whether and to what extent mobility devices substitute for personal assistance. It expanded on prior studies with the use of data from a recent national survey. This is critical given evidence of the changes in disability rates and use of accommodations over the past decades. Furthermore, we used a novel, joint modeling approach that enables us to directly measure the substitution effect. It allows for the likelihood of using devices and of receiving personal assistance to be jointly determined. Most previous studies have used single equation methods that do not allow for a structural determination of the relationship between the two. The use of this approach with a recent cohort of older adults will enhance our understanding of the relationship between mobility device use and personal assistance, two important but critically different forms of accommodation.

## Method

### Sample

We used the 2011 wave, baseline data, of the National Health and Aging Trend Study (NHATS), a nationally representative sample of Medicare enrollees 65 and older (Kasper & Freedman, 2014). The survey interviewed a total of 8,245 individuals with a response rate of 71%, including data on demographic characteristics, mobility conditions, physical and cognitive health, economic status, well-being, and quality of life. The current study used the sample of community-dwelling NHATS participants, excluding 468 nursing home residents and 168 participants in non-nursing residential facilities who did not complete interviews. In addition, 286 individuals with missing data on other covariates were also excluded from the analysis, with the majority of them missing body mass index (BMI) data. Finally, to study the individuals with mobility difficulty, 4,112 without mobility difficulty who did not use a mobility device or personal assistance were excluded. The sample included those who have mobility difficulty or those who used either a mobility device or personal assistance. Mobility difficulty was measured by asking whether individuals had difficulty moving inside, moving outside, or getting out of bed in the previous month. If they reported difficulties in any one of the activities, they were considered to have mobility difficulty. The final sample included 3,211 individuals.

### Measures

The two primary outcome variables about care arrangement were as follows: (a) any use of mobility device and (b) any use of personal assistance for mobility during the month before the baseline interview. Mobility device use was assessed using a yes/no question concerning use of a cane, walker, wheelchair, or scooter in the last month. To assess personal assistance, the survey asked respondents yes/no questions concerning whether in the previous month they had received personal help going outside their home or building, getting around inside, or getting out of bed. They were considered to use personal assistance if they received help with any of the mobility tasks.

Gender was measured dichotomously (male/female). The sociodemographic predictors included age, race/ethnicity (White, Black, Hispanic), education (less than high school, high school, some vocational training or college, and bachelor’s degree or higher), and income divided into five groups (<US$14,000; US$14,000-US$21,999; US$22,000-US$35,999; US$36,000-US$59,000; >US$60,000).

We also included health insurance, having Medicare supplemental insurance and having Medicaid (yes/no). Social/Physical Environment was assessed with questions concerning living alone (yes/no) and living in a retirement community (yes/no). In addition, physical environment was measured with the inclusion of a question about the presence of stairs or a step (yes/no) at the entrance of the respondent’s home or building.

Physical and cognitive capacity and mental health measures also were used. A physical capacity score was computed using six pairs of tasks (walking three or six blocks, climbing 10 or 20 stairs, lifting and carrying 10 or 20 pounds, bending over or kneeling down, reaching overhead or placing a heavy object overhead, grasping small objects, or opening a sealed jar). Participants received a 1 or 2 depending on whether they could do only one or both tasks for each pair. The total number was summed (1-12), with higher values indicating greater physical capacity (Freedman et al., 2011). Probable and possible dementia were determined based on the NHATS classification scheme, which consisted of self-reported physician diagnosis of Alzheimer’s disease or dementia, the AD8 dementia screening interview (administered to proxy respondents), and tests of memory, orientation, and executive function (Kasper, Freedman, & Spillman, 2013). Depression measurements were based on questions from the two-item depression screener (Patient Health Questionnaire [PHQ]-2) that generated a symptom score ranging from 0 to 6, with depression at >3 (Kroenke, Spitzer, & Williams, 2003).

We used physical impairment and health variables found in previous research to be associated with mobility device use (Peterson, Meng, Dobbs, & Hyer, 2016). These included participant reports (yes/no) of whether they had pain, balance problems, limited lower body strength, or limited upper body strength. Hospitalization was measured with a question concerning whether participants had been hospitalized overnight in the past 12 months. BMI was calculated using measured height and weight (BMI = weight in kilograms divided by height in meter squared), with participants categorized as underweight (BMI < 18.5), overweight (25 ≤ BMI < 30), or obese (BMI ≥30), with the default category of normal weight. Comorbidities related to mobility device use were measured with yes/no questions concerning whether participants had been medically diagnosed with stroke, arthritis, osteoporosis, or diabetes.

### Statistical Analysis

All statistical analyses were performed using STATA version 13 (Stata Corp., College Station, TX). Bivariate analyses were used to examine the independent variables by accommodation (mobility device alone, personal assistance alone, both, and neither). We then used recursive bivariate probit models to jointly estimate the effect of covariates on the likelihood of using mobility devices and personal assistance (Greene, 2003). Bivariate probit models are suitable for the joint modeling of two dichotomous dependent variables that are correlated. An additional benefit of the bivariate probit model is that the use of devices and personal assistance is not assumed to occur in any order. This approach has been used in economic, health outcomes and other studies (Gandelman, 2009; Liu, Chen, Chan, & Chen, 2015). The model consists of two equations. To obtain an estimate of the structural effect of mobility device use on personal assistance use, the dependent variable of the second equation (device use) was entered into the first equation (personal assistance) as an independent variable, thereby linking the two equations to form a recursive model. This joint modeling approach helps to answer the question of whether mobility devices substitute for personal assistance. Independent variables in the present study included sociodemographics, environment, physical/cognitive capacity, mental health status, and physical impairments and health conditions.

## Results

The final sample included 3,211 individuals with an average age of 80.4 years (*SD* = 8.2; range = 65-106); 64.7% were female. The sample included four groups of individuals based on their use of a mobility device and/or personal assistance. Of the whole sample, 37.3% used mobility devices only, 7.9% used personal assistance only, 30.8% used both, and 24.1% used neither. Table 1 displays the individual characteristics of each group, showing that participants differ based on the profile of use. Those who used a mobility device only were more likely to have Medicare supplemental insurance (58.2%), to live alone (45.6%), and to be obese (39.5%). Those who used personal assistance only were more likely to have probable dementia (46.1%) and to have depression (58.7%). Those who used both were more likely to have physical impairments, low balance (74.1%), low lower body strength (76.1%), and low upper body strength (60.2%). They also were more likely to have been hospitalized (47.7%). As expected, those who used neither were younger, with higher income, and a low proportion of Black participants. This group also was less likely to be on Medicaid and to be healthier. Interestingly, a large proportion of those who used neither accommodation reported pain (72.8%) and having arthritis (67.2%).

**Table 1.** Baseline Characteristics of Sample Population, by Usage, National Health, and Aging Trends Study, 2011.

	Mobility device only	Personal assistance only	Both	Neither
	(*n* = 1,198, 37.3%)	(*n* = 254, 7.9%)	(*n* = 988, 30.8%)	(*n* = 771, 24.1%)
Sociodemographics				
Age	80.6	79.2	83.5	76.4
Female	60.5%	74.0%	73.8%	56.5%
Education	2.3	1.9	2.0	2.4
Income	2.6	2.3	2.1	2.9
Black	27.0%	24.0%	28.4%	21.3%
Hispanics	5.3%	17.3%	8.0%	6.1%
Medicare supplemental	58.2%	39.4%	48.1%	56.9%
Medicaid	19.5%	31.9%	34.2%	15.2%
Social/physical environment				
Lives alone	45.6%	23.2%	34.0%	30.7%
Lives in retirement community	20.1%	13.4%	15.0%	10.1%
Outside stairs	66.3%	75.6%	77.3%	66.2%
Physical and cognitive capacity				
Physical capacity	5.9	5.0	2.6	8.8
Possible dementia	18.6%	13.0%	17.2%	13.4%
Probable dementia	12.9%	46.1%	40.7%	7.9%
Depression	33.6%	58.7%	52.3%	38.7%
Physical impairment				
Pain	71.7%	65.0%	74.3%	72.8%
Low balance	50.6%	54.7%	74.1%	39.0%
Low lower body strength	59.6%	53.9%	76.1%	55.1%
Low upper body strength	39.3%	44.9%	60.2%	42.2%
Health conditions				
Hospitalization	32.7%	35.4%	47.7%	21.3%
Underweight	1.8%	9.5%	6.7%	2.3%
Overweight	31.9%	28.0%	32.8%	35.9%
Obese	39.5%	24.0%	28.6%	34.5%
Stroke	16.0%	18.1%	26.0%	11.7%
Arthritis	73.9%	59.4%	75.2%	67.2%
Osteoporosis	25.8%	26.8%	32.7%	21.5%
Diabetes	32.0%	27.2%	34.7%	27.0%

Table 2 displays the results from the recursive bivariate probit model predicting the joint likelihood of using a mobility device and/or personal assistance. The likelihood ratio test comparing the likelihood of the joint bivariate models with the sum of the log likelihoods for the univariate probit models was statistically significant at the .001 level, suggesting that the bivariate probit model was appropriate in modeling the joint distribution of the outcome variables. Results showed that using a mobility device was significantly associated with a 50% lower likelihood of receiving personal assistance (95% confidence interval [CI] = [0.29, 0.86]).

**Table 2.** Predictors of Personal Assistance and Mobility Device Use, Recursive Bivariate Probit Model.

Variables	Personal assistance			Mobility device use		
	Odds ratio	*p* value	95% CI	Odds ratio	*p* value	95% CI
Sociodemographics						
Age	0.83	.001	[0.74, 0.93]	0.91	.131	[0.80, 1.04]
Female	**1.32**	**<.001**	**[1.15, 1.52]**	**0.75**	**<.001**	**[0.67, 0.85]**
Education	1.04	.137	[0.99, 1.10]	1.08	.007	[1.02, 1.14]
Income	0.99	.771	[0.95, 1.04]	1.00	.885	[0.95, 1.04]
Black	0.92	.253	[0.81, 1.06]	1.24	.001	[1.09, 1.42]
Hispanics	1.06	.583	[0.86, 1.31]	0.76	.012	[0.62, 0.94]
Medicare supplemental	0.91	.107	[0.82, 1.02]	1.15	.013	[1.03, 1.28]
Medicaid	1.35	<.001	[1.18, 1.54]	1.07	.355	[0.93, 1.24]
Social/physical environment						
Lives alone	**0.72**	**<.001**	**[0.63, 0.82]**	**1.22**	**<.001**	**[1.08, 1.38]**
Lives in retirement community	0.91	.270	[0.78, 1.07]	1.05	.581	[0.89, 1.24]
Outside stairs	1.05	.437	[0.93-1.19]	0.81	.001	[0.72-0.92]
Physical and cognitive capacity						
Physical capacity	**0.83**	**<.001**	**[0.80, 0.85]**	**0.84**	**<.001**	**[0.83, 0.86]**
Possible dementia	1.08	.304	[0.93, 1.24]	1.05	.489	[0.91, 1.22]
Probable dementia	**1.70**	**<.001**	**[1.46, 1.98]**	**0.77**	**.001**	**[0.67, 0.89]**
Depression	**1.14**	**.030**	**[1.01, 1.29]**	**0.75**	**<.001**	**[0.67, 0.84]**
Physical impairment						
Pain	0.89	.071	[0.79, 1.01]	1.00	.939	[0.89, 1.14]
Low balance	**1.21**	**.001**	**[1.08, 1.35]**	**1.15**	**.020**	**[1.02, 1.29]**
Low lower body strength	**1.17**	**.012**	**[1.03, 1.31]**	**1.16**	**.016**	**[1.03, 1.31]**
Low upper body strength	0.99	.915	[0.88, 1.12]	0.81	<.001	[0.72, 0.91]
Health conditions						
Hospitalization	**1.41**	**<.001**	**[1.27, 1.57]**	**1.26**	**<.001**	**[1.13, 1.42]**
Underweight	1.33	.040	[1.01, 1.75]	0.77	.068	[0.59, 1.02]
Overweight	1.00	.983	[0.87, 1.14]	1.14	.053	[1.00, 1.30]
Obese	0.92	.251	[0.79, 1.06]	1.34	<.001	[1.16, 1.54]
Stroke	1.14	.052	[1.00, 1.30]	1.12	.113	[0.97, 1.30]
Arthritis	0.82	.002	[0.72, 0.93]	1.12	.061	[0.99, 1.27]
Osteoporosis	1.02	.742	[0.90, 1.15]	1.12	.077	[0.99, 1.27]
Diabetes	1.12	.064	[0.99, 1.25]	1.16	.015	[1.03, 1.30]
Mobility device	**0.50**	**.012**	**[0.29, 0.86]**	—	—	[—]

*Note.* Boldface type indicates a significant association (<.05) between the independent variable and both personal assistance and mobility device use. CI = confidence interval.

Predictors of personal assistance and mobility device use exhibited similarities and differences. Several variables affected both the outcome variables in the same direction. Those with greater physical capacity were less likely to receive personal assistance (odds ratio [OR] = 0.83, 95% CI = [0.80, 0.85]) and less likely to use a mobility device (OR = 0.84, 95% CI = [0.83, 0.86]). By contrast, those with a previous hospitalization were more likely to receive personal assistance (OR = 1.41, 95% CI = [1.27, 1.57]) and more likely to use a mobility device (OR = 1.26, 95% CI = [1.13, 1.42]), as were those with low balance and low lower body strength.

Other variables affected outcome variables in the opposite direction. Women were more likely to use personal assistance (OR = 1.32, 95% CI = [1.15, 1.52]), but were less likely to use mobility devices (OR = 0.75, 95% CI = [0.67, 0.85]). Likewise, those with probable dementia were more likely to use personal assistance (OR = 1.70, 95% CI = [1.46, 1.98]), but were less likely to use mobility devices (OR = 0.77, 95% CI = [0.67, 0.89]). Conversely, those who lived alone were less likely to use personal assistance (OR = 0.72, 95% CI = [0.63, 0.82]) and more likely to use mobility devices (OR = 1.22, 95% CI = [1.08, 1.38]).

Several other variables were significantly associated with the use of one accommodation but not with use of the other. For example, having Medicare supplemental insurance was significantly associated with a greater likelihood of using mobility devices. However, there was no substitution effect because the likelihood of receipt of personal assistance was not significant.

## Discussion

This study simultaneously examined the use of personal assistance and mobility devices, finding that among community-dwelling Medicare beneficiaries with mobility difficulties, those who used a mobility device were less likely to use personal assistance. These findings provide support for previous research indicating that assistive devices have the potential to substitute for personal assistance (Agree & Freedman, 2000; Allen et al., 2001; Anderson & Wiener, 2015; Hoenig et al., 2003).

A contribution of the present research is our finding that the multiple factors associated with personal assistance and mobility device usage exhibited important similarities and differences. Although we found evidence overall of a substitution effect, usage of one form of accommodation or another differed depending on participants’ conditions or situations. Those who lived alone were less likely to use personal assistance and more likely to use a mobility device, suggesting that devices may substitute for personal assistance with this group and may enable individuals who are alone to continue to live independently as they encounter mobility difficulties. These results support prior research concerning device use among those who live alone (Elliott, Painter, & Hudson, 2009).

By contrast, those with a previous hospitalization or physical impairment (low balance or low lower body strength) were more likely to use both personal assistance and a mobility device in the present research. Similarly, Agree and colleagues (2005) found a complementary relationship between device use and personal care for those with more activity of daily living limitations. It is possible that older adults with higher levels of illness or impairment may not be able to forego personal assistance, despite the use of a device. In addition, caregivers may be facilitating assistive device use for those receiving care at home to complete their care tasks more safely or efficiently, as Anderson and Wiener (2015) suggested. In keeping with our results concerning hospitalization or presence of an impairment, participants with higher physical capacity, who are likely to be healthier overall, were less likely to use both personal assistance and mobility devices.

We found different patterns with other groups of participants. Women were more likely to use personal assistance and less likely to use a mobility device. Prior research found that women were less likely than men to use a cane, and that receipt of personal assistance was a factor in the observed association (Peterson et al., 2016). Research also has found that the use of mobility devices, in particular canes and walkers, was aversive to women who linked it to the idea of becoming older and more vulnerable (Porter, Benson, & Matsuda, 2011). Depression also was associated with a greater likelihood of receiving personal assistance and a lower likelihood of using a mobility device. Other research has linked depression and device use, with Tomita, Mann, Fraas, and Stanton (2004) finding that depression was associated with the use of fewer devices. Resnik and colleagues (2009) reported in qualitative research a relationship between the need for mobility devices and feelings of depression, such that device use was considered to signal a weakness or deficit. These studies and others (Verbrugge, 2016) suggest there is a psychosocial aspect to the use of assistive devices, which may help explain the lesser likelihood of using mobility devices among some groups of participants. Some older adults may reject assistive devices, Anderson and Wiener (2015) suggested, because using them could result in less personal interaction with their caregivers. They also suggested the devices may not work equally well for everyone who needs them.

The latter suggestion may partly explain our additional finding that those with more severe cognitive impairment were more likely to receive personal assistance and less likely to use a mobility device. In their report on the technical difficulties of using some mobility devices, Bateni and Maki (2005) highlighted the need for new devices and device designs that place fewer cognitive demands on their users.

Overall, the studies concerning the factors associated with lesser device use suggested that for certain individuals, device use was aversive and/or difficult. These obstacles, however, could be overcome through education and training on the benefits of using devices, combined with efforts to create more usable devices or environments in which devices are easier to use. This is particularly important in light of previous findings that devices are uniquely beneficial in easing the difficulty of daily tasks (Verbrugge & Sevak, 2002). Increasing the use of assistive devices could increase the independence of those who need help with their daily activities and reduce the strain on caregivers and the demand for their services.

Some limitations should be noted in the present study. This is a cross-sectional analysis. Although our model controlled for some of the endogeneity, we cannot claim any causal relationships. In addition, although we controlled for health conditions and functional limitations, we were not able to account for all the effects of physical and functional decline on choice of accommodation. Longitudinal analysis is needed to study the dynamic change in choices of care arrangement and the underlying mobility and health conditions overtime. Another limitation is that we could not conduct analyses by specific device type using the joint modeling approach. The devices were not used mutually exclusively. Individuals in the study sample could have been combining the use of two or more of the four devices. However, other research has found evidence that type of device (e.g., cane or walker) does affect the likelihood of receiving personal assistance (Agree & Freedman, 2000; Allen et al., 2001). Future research assessing device use in more specific detail would produce important additional information about how to provide appropriate services to those with mobility difficulty. In addition, we did not use measures of the community environment (e.g., neighborhood disorder and safety) that may affect the availability of personal assistance. Such variables should be included in future work concerning devise use and personal assistance. It is notable that our results do show a device substitution effect for those who may have less access to personal assistance because they live alone.

In all, using a nationally representative sample of community-dwelling Medicare participants and a joint modeling approach, the current study found evidence that mobility devices substitute for personal assistance. Similar findings have emerged from studies using older data. Recent research has found that the proportion of older adults using mobility devices is increasing (Gell, Wallace, LaCroix, Mroz, & Patel, 2015). Together, these findings suggest that individuals are using assistive devices to maintain their independence, which is important given concerns about the availability of caregivers for older and disabled adults. Nevertheless, our results show that many others continue to rely on personal assistance to accommodate their mobility difficulties. This contributes to our understanding of some of the factors underlying the use of assistive devices and/or personal assistance for mobility. Greater knowledge of these factors may enable health care providers to better target recommendations for accommodations. In addition, research is needed into whether and how reluctance, aversion, or inability to use a mobility device can be modified to further increase independence among those with mobility disabilities.
